# Threshold for maximal electroshock seizures (MEST) at three developmental stages in young mice

**DOI:** 10.24272/j.issn.2095-8137.2019.038

**Published:** 2019-05-18

**Authors:** Cheng Xiang, Zhi-Na Li, Tian-Zhuang Huang, Jing-Hui Li, Lei Yang, Jing-Kuan Wei

**Affiliations:** 1 Faculty of Life Science and Technology, Kunming University of Science and Technology, Kunming Yunnan 650500, China; 2 First Affiliated Hospital of Kunming Medical University, Kunming Yunnan 650032, China; 3 Department of Neurosurgery, Kunming Children's Hospital, Kunming Yunnan 650021, China

DEAR EDITOR,

Early brain development after birth is extremely dynamic, suggesting that potential functional changes occur during this period. In this study, the maximal electroshock seizure threshold (MEST) was used to explore the electrophysiological variation among three developmental stages in young mice (no more than 5 weeks old). The induced electroshock seizure (ES) behavior of early postnatal mice (1–2-weeks old) differed from that during weaning (3 weeks old) and early puberty (4–5-weeks old). Thus, we further explored their respective characteristic responses to the ES parameters. When the stimulation current (SC) was limited to 4.0 mA, only the 1–2-week-old mice were induced to exhibit ES behavior at voltages of 30 V and 40 V, indicating they were more sensitive to maximal electroshock seizure (MES) (response to lower voltage). Surprisingly, however, they showed substantially lower mortality than the older groups under higher voltage conditions (60, 100, 160, and 200 V), suggesting better tolerance to the SC. We also found that when the current limit decreased to 3.5 mA, the 4–5-week-olds mice exhibited stable ES behavior with low mortality, while for 3-week-olds mice, the SC limit required to be reduced to 1.5 mA. In conclusion, our findings showed that neural sensitivity to MES was significantly different in young mice before puberty. Thus, greater attention should be given to distinguishing the developmental period of mice, especially in electrophysiological examination.

While the macroscopic layout of the brain is nearly complete by the end of pregnancy, it develops continuously at a high speed until prepuberty (Stiles & Jernigan, 2010). Recent magnetic resonance imaging studies have depicted structural change processes by brain volume (Gogtay et al., 2004; Knickmeyer et al., 2008) and fiber connection (Chen et al., 2016; Li et al., 2015) growth curves, suggesting that early brain development after birth is extremely dynamic (Li et al., 2013). In addition, many developmental mental disorders likely originate from developmental problems in preadolescents (Chen et al., 2017; Li et al., 2016). For example, it is estimated that 10.5 million children under 15 have active pediatric epilepsy, which is more than 10 times greater than that found in adults (Keezer et al., 2016).

Maximal electroshock seizure (MES) is an experimental paradigm that induces synchronous neural discharges in the brain through artificial current input (Kamei et al., 1978), and is used to induce acute epileptic behaviors (Fischer & Muller, 1988). However, there are limited reports on the application of MES in young mice to mimic childhood epilepsy. In this study, a MES threshold paradigm was applied to investigate electrophysiological variations in young mice less than five weeks old. Group information was summarized in Table 1. Behavioral expression in the mice included electroshock seizure (ES), death, and no response, which reflected their brain network states. Young mice less than 5 weeks old were divided into three groups: 1–2-week-, 3-week-, and 4–5-week-old groups, based on their physical features and activities. These groups corresponded to three critical developmental stages: i.e., early postnatal, weaning, and early puberty (Miao, 1997; Xu et al., 2011).

**Figure 1:**
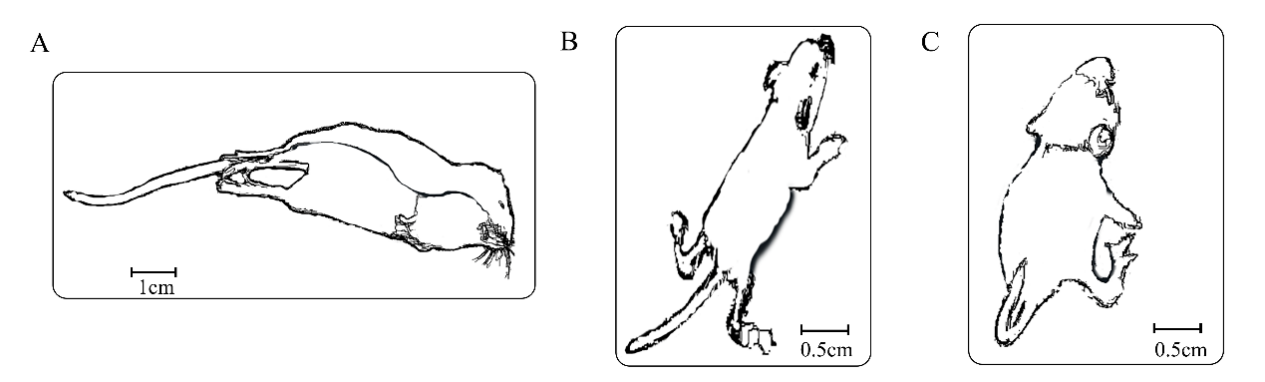
Electroshock seizure (ES) behavior at different developmental stages in mice

Usually, an electrical stimulus of the MES paradigm is delivered to adult mice (more than 6–week old) and is about 3–10 times higher than the individual electrical seizure threshold of the animal (Kamei et al., 1978; Murakami et al., 2007). The typical MES behaviors are: hind-limb extension, fall, and back rigidity, followed by foaming at the mouth and urinary incontinence (Ferraro et al., 1998, 2002)(Figure 1A). We found that seizure in 3–week-old and 4–5-week-old mice induced typical MES behavior as Figure 1A shown. Under the same conditions (i.e., SC limited to 3.5 mA and voltage of 80 V), the induced seizures of early postnatal mice (1–2 weeks old) were different; although urinary incontinence and mouth foaming were also observed, the seizures of 1–2-week-old mice did not include hind-limb extension and fall, with most limbs bending and convulsing (Figure 1B).

**Table 1:** Animals were grouped based on age and current limit in the MEST test

	**Current limited to 4.0 mA**	**Current limited to 3.5 mA**	**Current limited to 1.5 mA**	**Total (*n*)**
**1–2 weeks**	85	10	10	105
**3 weeks**	36	25	52	113
**4–5 weeks**	39	70	23	132
**Total (*n*)**	160	105	85	350

A: ES behavior of 3-week-old and 4–5-week-old mice. B: Typical ES behavior patterns of 1–2-week-old mice at low voltage (30–100 V, 4.0 mA); C: ES behavior patterns of 1–2-week-old mice at high voltage (120–200 V, 4.0 mA).

**Figure 2:**
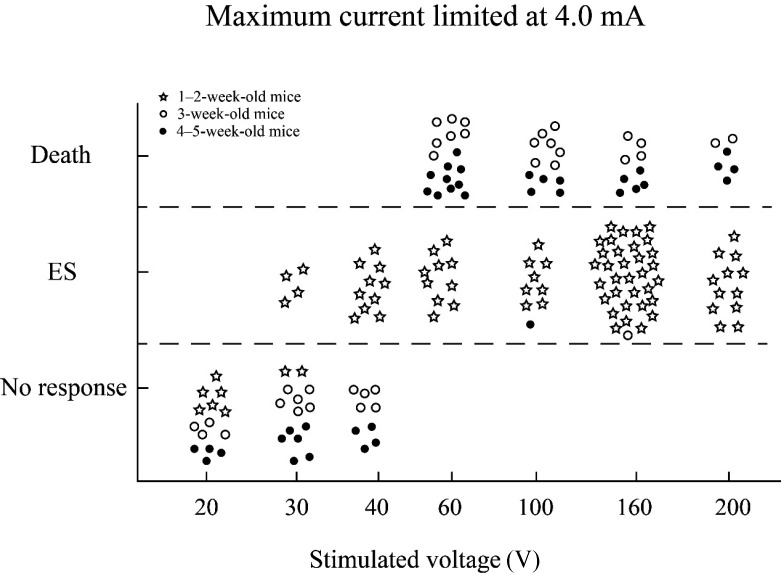
**Maximal electroshock seizure (MES) occurrence, no-response, and death voltage range of 1**–**2-week-old, 3-week-old, and 4**–**5-week-old mice at 4.0 mA**

These behavioral differences may be due to the sensitivity differences in nerves. Therefore, the mice were subjected to electrical stimuli with elevated parameters. Surprisingly, when the SC was limited to 4.0 mA and the voltage was 160–200 V, all 1–2-week-old mice survived the elicited ES behaviors. Under the above parameters, all behaviors exhibited by 1–2-week-old mice were consistent with typical opisthotonus, which differs from the previous stimulus conditions. The MES behaviors in 1–2-week-old mice included bilateral forelimb clonus, tail stiffness, and bending (Figure 1C). In contrast, under the same conditions (i.e., 4.0 mA and 160–200 V), all but one of the 3–week-old and 4–5-week-old mice died after rigidity seizures (Figure 2).

When the maximum output current was limited to 4.0 mA, death, MES, and no response were in response to constant voltage electrical stimulation. ES: Electroshock seizure.

Based on the above results, a series of stimuli was given to the three groups of young mice to determine the optimal range of parameters for inducing ES behavior.

As shown in Figure 2, when the SC was limited at 4.0 mA and the voltages were 30 V and 40 V, ES behavior was observed in 1–2-week-old mice (87.5%, *n*=16) but not in the other groups, indicating that the 1–2-week-old mice were more susceptible to electrical stimulation than the older groups (Figure 2). This age is equivalent to the human breastfeeding stage before 2 years old. Furthermore, this result is consistent with the clinical epidemic status of epilepsy, a brain disease with abnormal synchronous neural discharge of the cerebral cortex, thus indicating that the early postnatal brain is more sensitive to electrical stimulations.

Interestingly, at voltages of 60, 100, 160, and 200 V (Table 2), 100% of 1–2-week-old mice survived after ES behavior occurrence (63/63). In contrast, 95.6% of 3-week-old and 4–5-week-old mice died (44/46), demonstrating a substantially higher mortality than the youngest group (*P*<0.000 1). This indicated that early postnatal mice better endured electrical stimulation than early puberty mice, which has not been reported previously.

**Table 2:** **Responses of 1**–**2-week-old and 3**–**5-week-old mice under 4.0 mA and 60**–**200 V**

**Age**	**Number (*n*)**	**ES**	**Death**	***Chi*** **-square test**
**1–2 weeks**	63	100%	0	*P*<0.000 1
**3–5 weeks**	46	4.3%	95.6%	

Statistics were analyzed by *Chi*-square test. ES: Electroshock seizure.

**Figure 3:**
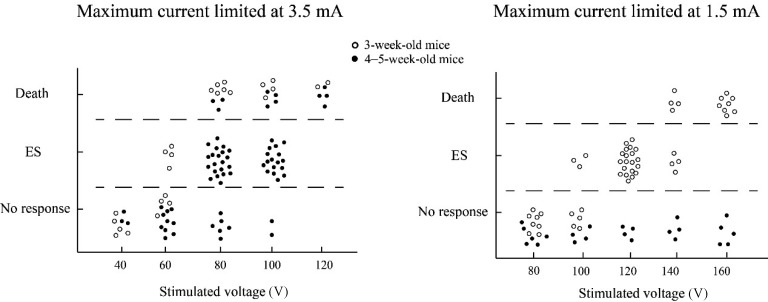
**Voltage range of 3-week-old and 4**–**5-week-old mice showing successful maximal electroshock seizure (MES), no-response, and death with current limited to 3.5 mA or 1.5 mA**

Electrical stimuli with reduced current were applied to explore the appropriate stimulus range for 3-week-old and 4–5-week-old mice. As shown in Figure 3A, when the SC was limited to 3.5 mA, 72% (38 in 53) of 4–5-week-old mice elicited ES at voltages of 80 V and 100 V. This was a relatively safe stimulation range in which to induce ES behavior of 4–5-week-old mice. For 3-week-old mice, however, only 50% (4 in 8) exhibited successful induction of ES behavior at 60 V. Furthermore, when the voltage increased to 80, 100, and 120 V, all 3-week-old mice died. When the SC was limited to 1.5 mA, 3-week-old mice exhibited ES behavior within a broad range of voltages (100–140 V). This was therefore considered a suitable range of the stimulus parameters, although 4–5-week-old mice demonstrated no responses under these conditions. Comparing the three groups, we determined that the behavioral responses of young mice in different periods under a MEST paradigm were different due to the different intensities of brain network connections. The stimulated parameter ranges eliciting MES for different groups of young mice were not continuous. Thus, based on our results, it appears that 3 weeks of age may be a turning point in nervous system development in mice.

When the maximum output current was limited to 3.5 mA (left) or 1.5 mA (right), death, MES, and no response of 3-week-old (circle) and 4–5-week-old mice (solid circle) were in response to constant voltage electrical stimulation. ES: Electroshock seizure.

To validate that the behaviors induced by electrical stimulation in all young mice were the same as epileptic seizures, an antiepileptic positive drug inhibitory experiment was conducted. Phenobarbital sodium, which is a commonly used treatment for epilepsy, was used here to inhibit the epidemiology of MES under an adult median effective dose (ED_50_) of 2.0 mg/kg. Results showed that it significantly inhibited the onset of ES behavior in 1–2-week-old (10/16), 3-week-old (7/13), and 4–5-week-old mice (4/8), as shown in Table 3. These results suggest that although the behavior and ranges of voltage and current that induced ES in young mice differed from that observed in adults (Ferraro et al., 2002), they were still indicative of epileptic episode and could be inhibited by antiepileptic drugs.

**Table 3:** Inhibition of phenobarbital sodium on MES behavior

**Group**	**Treatment**		**Total (MES/Anticonvulsant)**	**Inhibition (%)**
**1–2 weeks**		ES	9 (9/0)	62.5 (*P* ^#^=0.008 8)
		ES+PB	16(6/10)	
**3 weeks**		ES	12(12/0)	53.8 (*P*=0.005 2)
		ES+PB	13(6/7)	
**4–5 weeks**		ES	16(16/0)	50 (*P*=0.006 6)
		ES+PB	8(4/4)	

ES: Electroshock seizure. PB: Phenobarbital sodium, which was administered via intraperitoneal injection at a dose of 2.0 mg/kg. ^#^: Statistics were analyzed by *Chi*-square test.

The present study used the MEST paradigm to explore neurophysiological differences in three developmental stages before puberty in mice. We found that seizure was induced in all three groups of young mice (less than 5 weeks of age) soon after electrical stimulation. The 1–2-week-old mice exhibited both forelimb and tail stiffness with mild convulsions, whereas the other two groups displayed hind-limb extension, fall, and back rigidity. All young mice foamed at the mouth during seizure and experienced urinary incontinence, but recovered to normal activity after 2–5 s. In addition to differences in seizure behavior, the three age groups demonstrated different behavioral outcomes under electrical stimulation. For example, 3-week-old mice showed significantly higher mortality when the SC was 4.0 and 3.5 mA. Only when the current was limited to 1.5 mA could they survive after induction of MES, whereas, the mice in the 4-5-week group showed no responses to electrical stimulations. The optimal MES stimulation conditions in 4–5-week-old mice in this study are similar to those of adult rats in previous studies (Ferraro et al., 2002). Unexpectedly, we found that not only did 1–2-week-old mice experience induced seizures at lower voltage, but they also survived at higher voltage stimulation.

Seizures induced by electroshock are one of the two most widely studied animal models of generalized epilepsy, the other being pentylenetetrazol (PTZ) administration (Loscher, 2011). Accumulated evidence implicates structures of the brainstem as being involved in both kinds of experimental seizures. Stimulation of the midbrain reticular formation induces motor seizures in cats, rats, and rabbits (Kreindler et al., 1958). The substantia nigra also seems to play an important role in mediating seizure discharge release. The seizures (tonic hindlimb extension behaviors of 3- and 4–5-week-old mice in the present study) induced by MES can be prevented by lesions of the substantia nigra in rats (Garant & Gale, 1983). Furthermore, injections of GABA agonist muscimol and opiates into the same region can also prevent MES-induced seizures (Iadarola & Gale, 1982). However, lesions in the mesencephalic reticular formation can antagonize the production of PTZ-induced convulsions (Jinnai et al., 1969). Moreover, seizures induced by PTZ administration rather than MES can be protect by bilateral diencephalic lesions. (Mirski & Ferrendelli, 1986). It is not yet clear whether the diencephalon and substantia nigra are parts of a single complex neuroanatomical network mediating experimental seizures or whether they belong to two separate independent pathways for propagation of different types of seizures. As mice reach sexual maturity at 6–7 weeks of age, these young mice possibly correspond to different human developmental periods: 1–2 weeks corresponds to a breastfeeding infant period, 3 weeks corresponds to the childhood weaning period, and 4–5 weeks corresponds to early puberty (Miao, 1997). In the new postnatal brain, the neural fiber connection is involved in rapid synaptic formation and redundant cutting. For 1–2-week-old mice, the connection state of the diencephalic and mesencephalic structures differs from that of the older group, which may be the cause of specific seizure phenotypes and greater resilience to electrical stimulation. Previous investigations have found that adult mice die from MES due to respiratory arrest (Buchanan et al., 2014). The high mortality of the 3-week-old mice may also be due to changes in the state of connection in the brainstem, resulting in increased susceptibility to inhibition of the respiratory center.

As pediatric epilepsy is one of the most vulnerable diseases in children (Keezer et al., 2016), the MEST paradigm was used to explore the physiological variation in the prepuberty brain. Animal epilepsy models of ES are useful for investigating pathophysiological mechanisms and developing or evaluating new antiepileptic treatments. However, the development of pediatric epilepsy models is still challenging due to dynamic development in the immature brain. Yet, MES and intravenous pentylenetetrazol tests can be used to demonstrate the anticonvulsant properties of anti-epileptic drugs. The former acts as an acute seizure model (Castel-Branco et al., 2009; Murakami et al., 2007), whereas the latter is a chronic model (Loyens et al., 2012). They can help to identify the effects of compounds on seizure spread and increase the seizure threshold, respectively. In this study, phenobarbital, an antiepileptic positive drug, was used to determine if the induced behaviors in 1–2-week-old mice were the same as epileptic seizures. The MES reactions were compared between mice with prior injection of phenobarbital (2.0 mg/kg) and the blank solvent control. Results showed that all MES behaviors in tested mice at the three developmental stages were significantly inhibited at similar intensity with phenobarbital, indicating that the MES behaviors of the mice were the same as those experienced during epileptic seizures.

Thus, physiological variation in the prepubertal brain to electrical stimulation at different developmental stages was found using the MEST paradigm, with the three age groups exhibiting different behavioral outcomes. The results from this study will improve our knowledge regarding early brain development and provide new evidence that neural pathology in children differs from that in adults, suggesting that the development of the brain from birth to adolescence is extremely dynamic. Thus, it is necessary to strictly divide the developmental stages of youth to obtain an appropriate animal model.
